# Salvianolate Ameliorates Osteopenia and Improves Bone Quality in Prednisone-Treated Rheumatoid Arthritis Rats by Regulating RANKL/RANK/OPG Signaling

**DOI:** 10.3389/fphar.2021.710169

**Published:** 2021-09-06

**Authors:** Xiang Gao, Qingyun Wu, Xinle Zhang, Jia Tian, Dahong Liang, Yalin Min, Jiaqi Lu, Xuemei Zhang, Liao Cui, Bilian Xu, Yanzhi Liu

**Affiliations:** ^1^ Department of Pharmacology, Guangdong Key Laboratory for Research and Development of Natural Drug, Guangdong Medical University, Zhanjiang, China; ^2^ Stem Cell Research and Cellular Therapy Center, The Affiliated Hospital of Guangdong Medical University, Zhanjiang, China; ^3^ Department of Pharmacy, Yangjiang People’s Hospital, Yangjiang, China; ^4^ Clinic Research Institute of Zhanjiang, Affiliated Central People’s Hospital of Zhanjiang of Guangdong Medical University, Zhanjiang, China

**Keywords:** rheumatoid arthritis, osteoporosis, salvianolate, glucocorticoid, collagen-induced arthritis, rats

## Abstract

Rheumatoid arthritis (RA) is closely associated with periarticular osteopenia and leads to a high risk of generalized osteoporosis. Although glucocorticoid (GC) treatment ameliorates joint degradation and manages inflammation in RA, GC application may induce further bone quality deterioration in RA patients. Current treatments for RA lack relevant strategies for the prevention and treatment of osteopenia in RA. In this study, we aimed to investigate whether salvianolate treatment ameliorated osteopenia in prednisone-treated RA rats. Lewis rats with collagen-induced arthritis (CIA) were administered prednisone (PDN) or PDN plus salvianolate (PDN+Sal) treatment for 90 days. The effects of Sal were investigated in PDN-treated CIA rats. To further evaluate the effects of Sal under inflammatory conditions, we investigated the effects of Sal treatment on the TNF-α-induced inflammatory response in MC3T3-E1 osteoblasts. Bone histomorphometry, bone mineral density (BMD), bone biomechanical properties, micro-computed tomography (micro-CT), immunohistochemistry, RT-PCR and western blot analyses were performed to evaluate the effects of Sal. The results demonstrated that RA induced bone loss and bone quality deterioration, with high bone turnover in CIA rats. PDN+Sal treatment significantly increased BMD and trabecular/cortical bone mass, suppressed inflammation, and improved bone biomechanical properties compared to CIA control and PDN treatment. PDN+Sal treatment significantly suppressed bone resorption and the RANKL and RANKL/OPG ratios compared to PDN. PDN+Sal and PDN treatment significantly inhibited TNF-α by 82 and 83%, respectively, and both suppressed inflammation in CIA rats. However, there was no significant difference between PDN+Sal and PDN treatment alone in regard to bone formation parameters or the management of inflammation and arthropathy. Sal significantly increased Osterix, OPN, and Col1a1 while decreasing RANKL, TRAF6, and TRAIL gene in TNF-α-induced MC3T3-E1 osteoblasts. Sal significantly increased Osterix, OPN and RUNX2 while decreasing NF-κB, TRAF6 and IL-1β protein in TNF-α-induced MC3T3-E1 osteoblasts. The results suggested that salvianolate treatment ameliorated osteopenia and improved bone quality in prednisone-treated RA rats, and the potential mechanism may be related to the regulation of the RANKL/RANK/OPG signaling pathway, TRAIL-TRAF6-NFκB signal axis, and downregulation of inflammatory cytokines. Salvianolate could be used as a promising supplemental therapeutic strategy to ameliorate osteopenia and improve bone quality in GC-treated RA patients.

## Introduction

Rheumatoid arthritis (RA) is a chronic inflammatory disease that often leads to severe joint damage, disability and even death (Guler-Yuksel et al., 2010). Bone loss and bone erosion around joints are serious issues in RA. RA is closely associated with periarticular osteopenia and leads to a high risk of generalized osteoporosis (Fassio et al., 2016; Okano et al., 2017; Coury et al., 2019). Current treatments for RA, such as nonsteroidal anti-inflammatory drugs (NSAIDs), disease-modifying antirheumatic drugs (DMARDs), biologics and glucocorticoids (GCs), have limited bone protection effects and even accelerate bone loss around joints or induce generalized osteopenia. GCs play an important role in the treatment of severe RA patients. Their potent anti-inflammatory effect significantly ameliorates RA symptoms; however, their potential side effects (e.g., GC-induced osteoporosis, GIOP) significantly limits the application of GCs in RA. Secondary osteoporosis is one of the major problems associated with long-term GC therapy. Bone mineral density (BMD) seemed to increase immediately in response to GC therapy but was ultimately reduced at a later time (Earp et al., 2008). Another report indicated that GCs (dosage ≥ 7.5 mg/day) were related to treatment failure in osteoporotic patients with RA (Wen et al., 2016). Concerns about these effects have prompted the development of new strategies to prevent bone loss and the deterioration of bone quality induced by RA or RA treatment with GCs.

*Salvia miltiorrhiza Bunge* is a traditional Chinese medicine that is widely used in clinical practice for the prevention and treatment of cardio-cerebral vascular diseases. Previous studies have shown that *Salvia miltiorrhiza* has anticoagulant, vasodilatory, and anti-inflammatory effects and other activities (Ji et al., 2000; Adams et al., 2006; Li et al., 2018; Jiang et al., 2019; Ren et al., 2019). *Salvia miltiorrhiza-*extracted cryptotanshinone significantly ameliorated inflammation and joint destruction in rats with collagen-induced arthritis (Wang et al., 2015). *Salvia miltiorrhiza* injection decreased the protein expression of NF-κB and TNF-α in RA rats (Liu et al., 2015). In addition, our previous studies demonstrated that bioactive components of *Salvia miltiorrhiza* prevented bone loss in prednisone-treated rats by stimulating osteogenesis, inhibiting bone absorption and suppressing adipogenesis in the bone (Cui et al., 2009; Cui et al., 2012; Yang et al., 2016). Salvianolate contains the total polyphenols extracted from *Salvia miltiorrhiza*. Salvianolate injection has been approved by the China SFDA for coronary heart disease treatment since 2005 (Approval number: Z20050248). Accordingly, salvianolate injection has been used for coronary heart disease treatment in the clinic for more than 15 years in China. It has been used in clinical practice for the treatment and prevention of cardio-cerebral vascular diseases, and its safety profile is well established in China (Chen et al., 2016; Zhang et al., 2016; Dong et al., 2018; NanZhu et al., 2018; Shen et al., 2020; Chai et al., 2021). Our previous study showed that salvianolate attenuated osteogenic inhibition and suppressed hyperactive bone resorption, which lead to the recovery of bone mass and bone mechanical properties in prednisone-treated lupus mice (Liu et al., 2016). However, whether salvianolate can ameliorate bone loss in RA or GC-treated RA individuals is still unknown. Since GCs are potent anti-inflammatory agents, it would be greatly beneficial if the burden of side effects of GC treatment could be reduced, especially the side effects involving the bone. Based on previous studies, we speculated that salvianolate might ameliorate osteopenia and provide joint protection in GC-treated RA individuals. In this study, we aimed to investigate the combined effect of prednisone and salvianolate on preventing joint damage and systemic bone loss in a CIA rat model.

## Materials and Methods

### Drugs and Reagents

Bovine type II collagen (Collagen II) was purchased from Sichuan University (Chengdu, China). Incomplete Freund’s adjuvant (IFA) was purchased from Sigma-Aldrich, China. Prednisone acetate was obtained from Guangdong South China Pharmaceutical Group Co., Ltd. (Guangdong, China). The salvianolate commercial injection product was purchased from Shanghai Green Valley Pharmaceutical Co., Ltd. (Shanghai, China). Salvianolate injection has been approved by the China SFDA for coronary heart disease treatment since 2005 (Approved number: Z20050248). Accordingly, salvianolate injection has been used for coronary heart disease treatment in the clinic for more than 15 years in China. According to the manufacturer’s instructions, one unit of salvianolate injection (50 mg) contained 40 mg salvianolic acid B magnesium salt (Magnesium lithospermate B, CAS. No: 122021-74-3 Supplementary Figure S1). The certificate of analysis and HPLC analysis were provided by manufacturer (Supplementary Figures S2, S3).

### Experimental Protocols

Forty-three female 6-week-old Lewis rats weighing 122 ± 12 g were obtained from Beijing Vital River Laboratory Animal Co. Ltd. (Beijing, China) [permit number: SCXK (Beijing) 2012-001]. All rats were housed in the animal facility of Guangdong Medical University for 2 weeks prior to the study. All protocols followed the Principles of the Care and Use of the National Laboratory Animal Monitoring Institute of China and were approved by the Academic Committee on the Ethics of Animal Experiments of Guangdong Medical University, Zhanjiang, People’s Republic of China, Permit Number: SYXK (GUANGDONG) 2008-0007. Forty-three 8-week-old female Lewis rats weighing 138 ± 13 g were randomly divided into two groups: a control (CON) group included seven rats, and the remaining 36 rats were used to induce the CIA rat model. The CIA rat model was induced as previously described (Mohammad Shahi et al., 2012). Briefly, rats were injected intradermally on the back at the base of the tail with 0.4 ml of collagen II emulsion (1.5 mg/ml). The same immunization procedure was performed again 1 week later to strengthen arthritis induction. Rats in the CON group were administered 0.4 ml acetic acid (0.05 mol/L) as a control. Four weeks after immunization, twenty-one CIA model rats had been successfully established (arthritis index≥4) for further study. The twenty-one CIA rats were randomly divided into three groups with seven rats per group: 1. the CIA rat group; 2. The prednisone-treated CIA rat group (CIA+PND); and 3. The prednisone and salvianolate combination treatment CIA rat group (CIA+PND+Sal). Rats in the CON and CIA groups were administered saline as a control, while rats in the CIA+PND and CIA+PND+Sal groups were treated with prednisone at 4.5 mg/kg/day (gavage) or/and salvianolate at 20 mg/kg/day (intraperitoneal injection). Drugs were administered every day after the CIA model was established, and the treatment continued for 90 days. On the 14th, 13th, fourth, and third days before the end point, all rats received subcutaneous injections of calcein (7 mg/kg) for *in vivo* bone formation fluorescence labeling. Rats were sacrificed by cardiac puncture under anesthesia.

### Bone Mineral Density and Biomechanical Property Analysis

The left femurs were collected and used for BMD analysis with a dual-energy X-ray absorptiometry system (DEX) (QDR-4500A, Hologic Inc. Bedford, MA, United States). After BMD analysis, the femurs were used for bone mechanical property analysis with a three-point bending test using an MTS Mini Bionix testing system (Mini Bionix 858, MTS System Co., Eden Prairie, MN, United States). Samples were tested with a 15-mm span at a speed of 0.01 mm/s. Force (F) and deflection (D) were automatically recorded in the system. The elastic load, maximum load, break load and stiffness coefficient were calculated for each sample.

### Micro-computed Tomography Analysis

The left distal femurs were collected for micro-CT analysis using a Viva CT40 analysis system (Scanco Medical, Zurich, Switzerland). The scan region was set to 500 slices with a 9.50-mm thickness in the distal femur, an integration time of 200 ms, energy of 70 kVp and a current of 114 mA. The lower and upper threshold was set at 170–1,000. The regions of interest (cancellous bone between 1- and 4-mm distal to the growth plate-metaphyseal junctions of the distal femur) were chosen for analysis. Bone volume/total volume (BV/TV), trabecular number (Tb.N), trabecular separation (Tb.Sp), trabecular thickness (Tb.Th), connectivity density (Conn.D), and the structure model index (SMI) were calculated with the built-in analysis software provided by the vendor.

### Bone Histomorphometric Analysis

The right proximal tibial metaphysis (PTM) and the fourth lumbar vertebra (LV) were processed to generate nondecalcified sections. The samples were cut to expose the bone marrow cavity with an Isomet Low Speed Saw (Buehler, Lake Bluff, Illinois, United States) and fixed in 10% formalin for 24 h, followed by 70% ethanol fixation and gradient alcohol dehydration. They were embedded in methyl methacrylate for nondecalcified sectioning. Frontal sections of the PTM/LV were prepared at thicknesses of 5 and 8 μm. The 5-μm sections were stained with Masson-Goldner Trichrome for static histomorphometric analysis. The 8-μm sections without staining were used for dynamic histomorphometric fluorescence measurements.

The region of interest in the PTM was located between 1- and 4-mm distal to the growth plate-epiphyseal junction. A semiautomatic digitizing image analysis system (Osteometrics, Decatur, GA, United States) was used for bone histomorphometric analysis. Static histomorphometric parameters included the total tissue area, trabecular area, trabecular perimeter, and osteoclast number (Oc.N). Dynamic histomorphometric parameters included the single-labeled perimeter, double-labeled perimeter, and interlabeled width. These parameters were used to calculate the percentage of trabecular bone volume (BV/TV), Tb.N, Tb. Sp, Oc.N number, the percentage of the osteoclast surface perimeter (%Oc.S.Pm), the percentage of the osteoblast surface perimeter (%Ob.S.Pm), the percentage of the labeled perimeter (%L.Pm), mineral apposition rate (MAR) and bone formation rate (BFR) per unit of bone surface (BFR/BS).

For the midtibial diaphyseal cortical bone, cross sections of the tibial shaft (3 sections per site) were cut to a 200 μm thickness using a low-speed metallurgical saw. Then, sections were ground to a thickness of 20 μm and cover slipped for histomorphometric analysis. The total cross-sectional area, cortical area, marrow area, single- and double-fluorescently labeled perimeter and interlabeling width for both periosteal and endocortical surfaces were measured, and the percentage of cortical area (%Ct.Ar), the percentage of marrow area (%Ma.Ar), and bone formation rates for periosteal (P-BFR/BS) and endocortical surfaces (E-BFR/BS) were calculated. The abbreviations of the bone histomorphometric parameters used were recommended by the American Society for Bone and Mineral Research Histomorphometric Nomenclature Committee (Dempster et al., 2013). All histomorphometric parameters and procedures were in accordance with a previously published study (Liang et al., 2019).

### Histological Evaluation

Right hind ankle samples were collected and fixed in 10% formalin. Then, the samples were decalcified in 15% ethylenediaminetetraacetic acid (EDTA) solution before paraffin embedding. The tissues were prepared in 4 µm-thick paraffin sections, followed by staining with hematoxylin and eosin (H&E) and histological score evaluation (Sajti et al., 2004). The histological scores were calculated with a comprehensive histological scoring system as described previously (Wu et al., 2016).

### Enzyme-Linked Immunosorbent Assay

Blood was collected from cardiac puncture under anesthesia. The serum was separated and stored at −80°C before analyses. Serum levels of tumor necrosis factor-α (TNF-α, catalog number: E-EL-R2856c), interleukin-6 (IL-6, catalog number: E-EL-R0015c), receptor activator of nuclear factor-κB ligand (RANKL, catalog number: E-EL-R0841c), and osteoprotegerin (OPG, catalog number: E-EL-R3005) were detected by enzyme-linked immunosorbent assay (ELISA) kits (Elabscience Biotechnology Co., Ltd., Wuhan, China.) according to the manufacturer’s instructions.

### Immunohistochemical Analysis

Immunostaining for tumor necrosis factor-α (TNF-α), RANKL and osteoprotegerin (OPG) was performed on 4-μm decalcified right distal femoral sections. Briefly, all sections were deparaffinized in xylene, rehydrated, and washed in PBS. The sections were then heated for 20 min in citrate buffer (pH 6.0) at 95°C and incubated with an anti-tumor necrosis factor-α (TNF-α) monoclonal antibody (1:100, catalog number: ab199013, Abcam, United States), anti-RANKL polyclonal antibody (1:200, catalog number: BA1323, Wuhan Boster, China), and anti-Osteoprotegerin (OPG) polyclonal antibody (1:500, catalog number: ab73400, Abcam, United States) overnight at 4°C. Phosphate-buffered saline (PBS) was used as a negative control. Then, the sections were incubated with secondary antibodies for 30 min at 26°C. 3,3′-Diaminobenzidine (DAB) staining and hematoxylin counterstaining were performed to visualize the expression of biomarkers. Immunohistochemical analysis was performed with Image-Pro Plus 6.0 software to evaluate immunostaining.

### Western Blot Analysis

Left hind ankles were snap-frozen in liquid nitrogen and ground into powder. Tissue homogenates were prepared and analyzed by western blotting. Protein expression of RANKL and OPG was detected using an anti-RANKL polyclonal antibody (1:200, catalog number: BA1323, Wuhan Boster, China) and anti-OPG polyclonal antibody (1:3,000, catalog number: ab203061, Abcam, United States), respectively. An anti-β-actin antibody (1:2,000, Catalog number: AP80340, SAB, United States) was used as a control. IgG antibodies (Beyotime, China) were used as secondary antibodies. Quantification was performed using Gel-Pro analyzer 4 software.

MC3T3-E1 cells were treated with TNF-α (50 ng/ml) or TNF-α (50 ng/ml) + Sal (1 μM), and the cells were collected for western blot analysis. The osteogenesis-related biomarkers OPN, Osterix and RUNX2 were analyzed after treatment for 24 h, while NF-κB, TRAF6 and IL-1β were analyzed after treatment for 72 h. Cell proteins were extracted and separated by SDS-PAGE. Proteins were then transferred to polyvinylidene fluoride (PVDF) membranes. The membranes were blocked with 5% skim milk in TBST, followed by incubation with primary antibodies overnight at 4°C. OPN (1:1,000, catalog number: ab8448, Abcam, United States), Osterix (1:1,000, catalog number: ab22552, Abcam), RUNX2 (1:1,000, catalog number: 12556, Cell Signaling, United States), NF-κB (1:1,000, catalog number: sc-8008, Santa Cruz, United States), TRAF6 (1:1,000, catalog number: ab33915, Abcam, United States), IL-1β (1:1,000, catalog number: 12242, Cell Signaling, United States), α-tubulin (1:1,000, catalog number: sc-32293, Santa Cruz, United States), and phosphor-NF-κB (1:1,000, Cell Signaling, catalog number: 3033S, United States) primary antibodies were used. After washing with TBST three times, the membranes were incubated with a horseradish peroxidase-conjugated secondary antibody (1:5,000 dilution, Santa Cruz, United States) at room temperature for 1 h. The immunoreactive bands were visualized by using an Immobilon® western chemiluminescent HRP substrate (WBKLS0100, Millipore, United States). The protein band images were subjected to semiquantitative analysis using ImageJ software and normalized to α-tubulin. Western blots were repeated a minimum of three times.

### Quantitative RT-PCR Assay

MC3T3-E1 cells were treated with TNF-α or TNF-α+Sal for 24 h, and the cells were collected for RT-PCR analysis. Total RNA was isolated from cells with TRIzol Reagent and quantified by a microspectrophotometer. HiScript® III All-in-one RT SuperMix Perfect for qPCR (333-01, Vazyme, China) was used to generate reverse-transcribed cDNA. PCR was performed using TB GreenTM Premix Ex TaqTM II (RR820A, TaKaRa, Japan) with the corresponding primers (Table 1). Osteopontin (OPN), Osterix (Osx), Collagen 1a1 (Col1a1), receptor activator of nuclear factor-kappa B ligand (RANKL), TNF-related apoptosis-inducing ligand (TRAIL), and TNF receptor associated factor 6 (TRAF6) were analyzed by RT-PCR. The real-time PCR primers were synthesized by Shanghai Sangon Biotech, Shanghai, China.

**TABLE 1:** The biomarker primers for RT-PCR analysis.

No.	Genes	Primers
1	OPN-Forward	GAG​ATG​GAG​TCT​TGC​TCT​GTC​ACC
	OPN-Reverse	AGG​CGG​ATC​ACG​AGG​TCA​GG
2	Osterix-Forward	TCG​TCT​GAC​TGC​CTG​CCT​AGT​G
	Osterix-Reverse	CTG​CGT​GGA​TGC​CTG​CCT​TG
3	Col1a1-Forward	TGA​ACG​TGG​TGT​ACA​AGG​TC
	Col1a1-Reverse	CCA​TCT​TTA​CCA​GGA​GAA​CCA​T
4	TRAF6-Forward	AGG​AAT​CAC​TTG​GCA​CGA​CAC​TTG
	TRAF6-Reverse	TCG​CAC​GGA​CGC​AAA​GCA​AG
5	TRAIL-Forward	CCT​CAG​CTT​CAG​TCA​GCA​CTT​CAG
	TRAIL-Reverse	GTA​AGT​CAC​AGC​CAC​AGA​CAC​AGC

The PCR program was as follows: preheating at 95°C for 30 s, 95°C heating for 5 s, and 60°C heating for 34 s, followed by 40 cycles of 95°C heating for 15 s and 60°C heating for 1 min. A Roche Light Cycler Detection System (Roche Diagnostics, Mannheim, Germany) was used to record the fluorescence signal and convert it into numerical values for statistical analysis. Relative gene expression was calculated by employing the comparative CT method, and the expression of the target gene was normalized to that of the housekeeping gene glyceraldehyde 3-phosphate dehydrogenase (GAPDH). The experiment was repeated 3 times.

### Statistical Analysis

Data are presented as the means ± SD and were analyzed using SPSS software 17.0 (SPSS Inc., Chicago, IL, United States). Data were analyzed by one-way ANOVA followed by post hoc Fisher’s protected least significant difference test with the homogeneity of variance or Dunnett’s T3 test with the heterogeneity of variance. *p* < 0.05 was considered significant.

## Results

### The Micro-CT and BMD Analysis of the Femur

**FIGURE 1:**
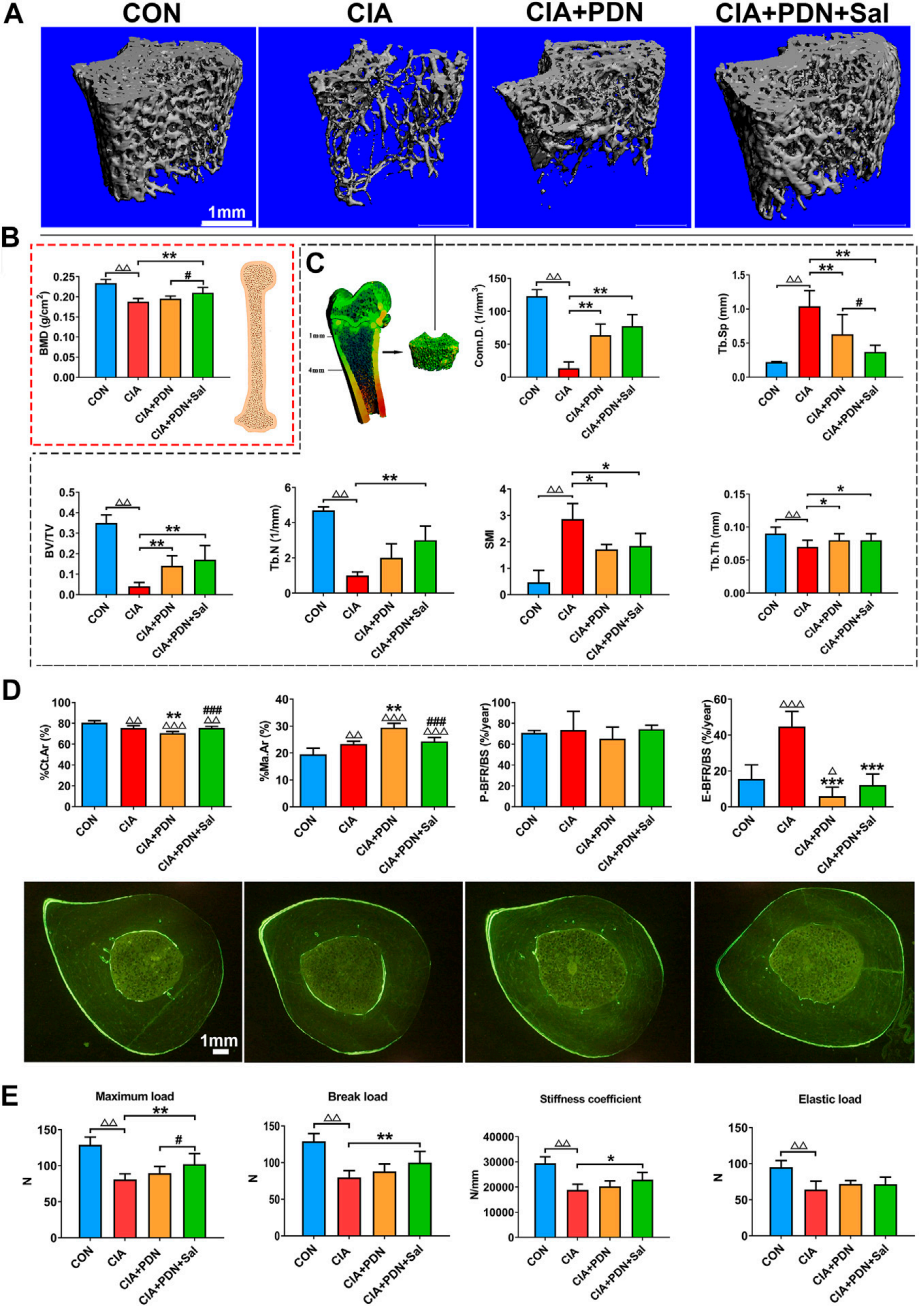
Representative micro-CT images **(A)**; micro-CT analysis of the distal femur metaphysis **(B)**; BMD analyses of the whole femurs **(C)**; bone histomorphometric analysis and representative images of the midshaft of the tibia **(D)** and bone biomechanical analysis of the femur **(E)** in different groups of rats. The femurs were collected for BMD analysis with a dual-energy X-ray absorptiometry system (DEX) and further used for biomechanical analysis. The distal femur metaphysis of rats was scanned and analyzed by a micro-CT system. The midshaft of the tibia were prepared for bone histomorphometric analysis. CON: healthy control rat group; CIA: collagen II-induced arthritis rat group; CIA+PND: prednisone-treated CIA rat group; CIA+PND+Sal: combination treatment with prednisone and salvianolate CIA rat group. ^ΔΔ^ *p* < 0.01 vs. CON, ^ΔΔΔ^ *p* < 0.001 *vs* CON; ^*^ *p* < 0.05, **p < 0.01, ***p < 0.001 vs. CIA; ^#^ *p* < 0.05, ^###^ *p* < 0.05 vs. CIA+PDN (*n* = 7).

Representative micro-CT analysis images are shown in Figure 1. Compared with the control group, BV/TV, Tb.N, Tb.Th and Conn. D were significantly decreased, while Tb. Sp and SMI were significantly increased, in the CIA group **(** Figure 1A **)**. BMD in the CIA group was significantly decreased by 19.6% compared to that in the control group (*p* < 0.01) (Figure 1B). Prednisone treatment in CIA rats (CIA+PDN) significantly increased BV/TV, Tb.Th, and Conn. D while decreasing Tb. Sp and SMI compared to those in CIA model rats(Figure 1C). Prednisone and salvianolate combination treatment in CIA rats (CIA+PDN+Sal group) significantly increased femur BMD and decreased Tb. Sp compared to those in the CIA and CIA+PDN group. Compared to the CIA model group, prednisone and salvianolate combination treatment significantly increased BV/TV, Tb.N, Tb.Th and Conn. D while significantly decreasing Tb. Sp and SMI (Figures 1A–C).

### Bone Biomechanical Property Analysis and Bone Histomorphometry Analysis of Cortical Bone

Histomorphometric analyses of the midshaft of the tibia demonstrated that %Ct.Ar in CIA group was significantly decreased, while %Ma.Ar and E-BFR/BS were increased in the CIA model group compared to those in the control. CIA+PDN significantly decreased %Ct.Ar and E-BFR/BS while increasing %Ma.Ar compared to that in the CIA control. CIA+PDN+Sal significantly increased %Ct.Ar, while decreasing %Ma.Ar compared to those in the CIA+PDN group. There was no significant difference in P-BFR/BS among the groups (Figure 1D).

The biomechanical properties of the femur (maximum load, break load, elastic load, and stiffness coefficient) in the CIA group were significantly decreased compared with those in the control group. Compared with those in the CIA group, the maximum load, break load, and stiffness coefficient were significantly increased in the CIA+PDN+Sal group. Compared to the CIA+PDN group, the CIA+PDN+Sal group had a significantly increased maximum load (Figure 1E).

### Bone Histomorphometry Analysis of the PTM

**FIGURE 2:**
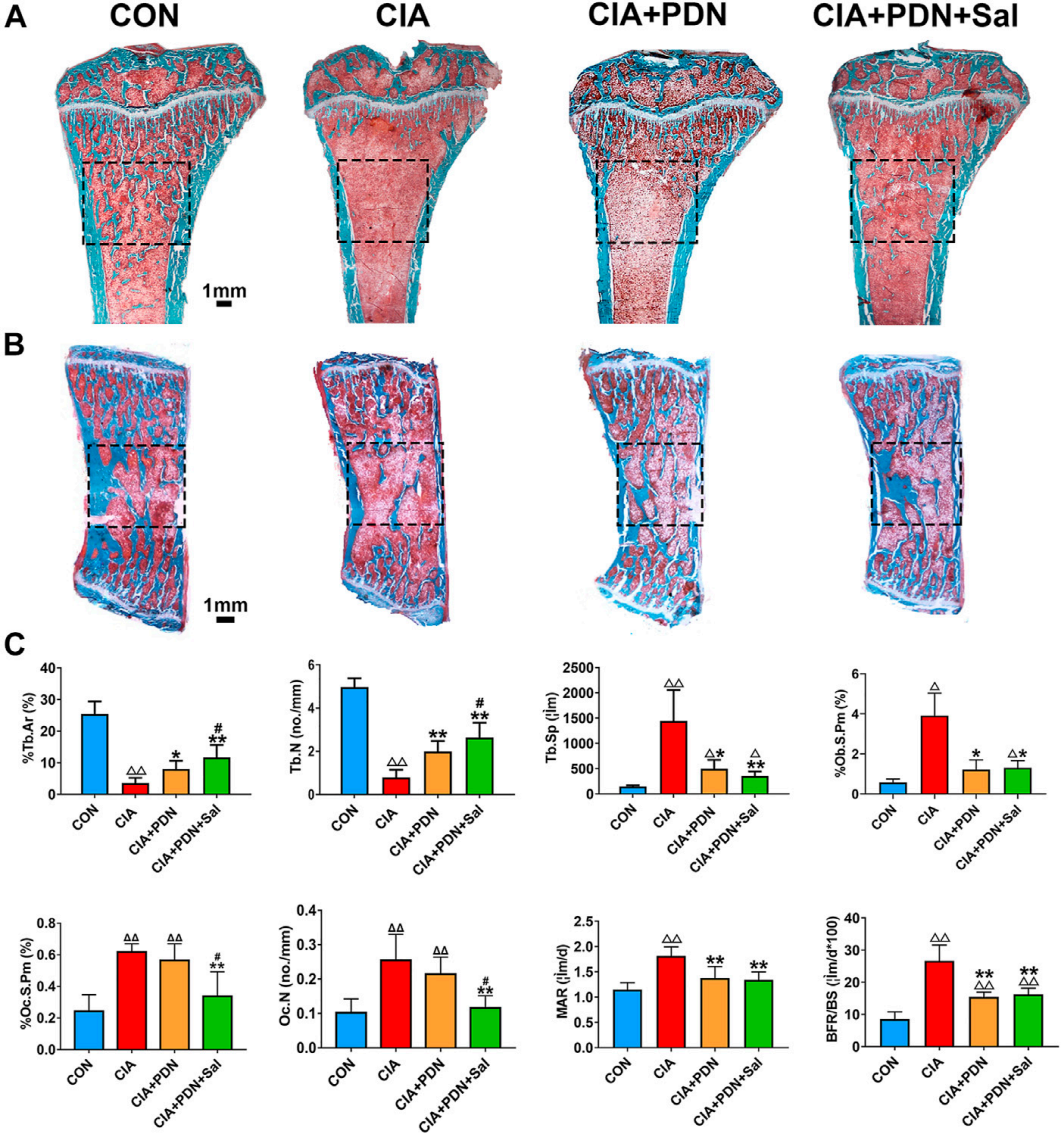
Representative images of the tibial metaphysis **(A)** and the lumbar vertebra **(B)** and bone histomorphometric analysis of the proximal tibial metaphysis **(C)** Nondecalcified tissue sections of the tibial metaphysis and lumbar vertebra were stained with Masson-Goldner trichrome. ^ΔΔ^ *p* < 0.01 vs. CON, **p < 0.05, **p < 0.01 vs. CIA, ^#^ *p* < 0.05 vs. CIA+PDN (*n* = 7).

Histomorphometric analysis demonstrated that %Tb.Ar and Tb.N of PTM in CIA rats were significantly decreased, while Tb. Sp, Oc. N, %Oc.S.Pm, MAR, %Ob.S.Pm and BFR/BS were significantly increased in the CIA model group compared to the control group. CIA+PDN significantly increased %Tb.Ar and Tb.N while decreasing Tb. Sp, Oc. N, %Oc.S.Pm, MAR and BFR/BS compared to those in the CIA control. CIA+PDN+Sal significantly increased %Tb.Ar, %Ob.S.Pm and Tb.N while decreasing Oc. N and %Oc.S.Pm compared to those in the CIA group and CIA+PDN group. Combination treatment with CIA+PDN+Sal did not increase MAR or BFR/BS compared to those in the CIA+PDN group (Figures 2A,C). The histomorphometric images of the lumbar vertebra showed the same trend as the PTM (Figure 2B).

### Histological and Immunohistochemistry Analyses

**FIGURE 3:**
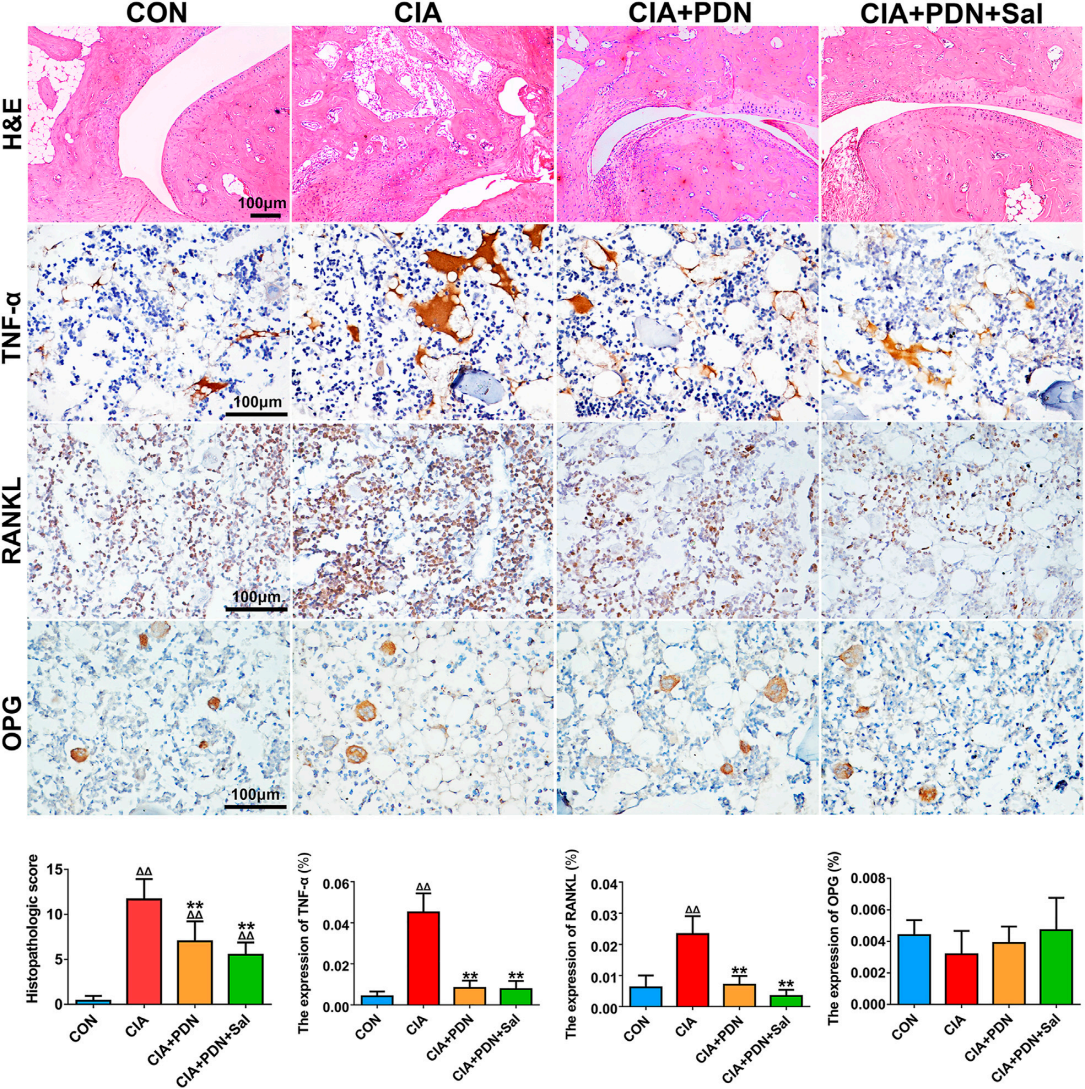
Histological and immunohistochemical analyses of ankle joints in different groups of rats. Paraffin sections of the ankle joint were prepared for H&E staining and immunohistochemical staining of TNF-α, RANKL, and OPG. The expression of biomarkers was evaluated by semiquantitative analysis with Image-Pro Plus software. ^ΔΔ^ *p* < 0.01 vs. CON, **p < 0.01 vs. CIA, ^#^ *p* < 0.05 vs. CIA+PDN (*n* = 7).

As shown in Figure 3, the histopathologic scores of ankle sections in the CIA group were significantly increased compared to those in the control group. The histopathologic scores were significantly decreased in the CIA+PDN and CIA+PDN+Sal groups compared to the CIA and control groups. Although CIA+PDN+Sal decreased histopathologic scores compared to CIA+PDN, there was no significant difference between the two groups.

Immunohistochemistry analyses demonstrated that the expression of TNF-α and RANKL in CIA rats was significantly increased compared to that in the control group. Compared with that in the CIA group, the expression of TNF-α and RANKL was decreased in the CIA+PDN group and CIA+PDN+Sal group. OPG expression in the CIA group was decreased compared to that in the control group. The CIA+PDN+Sal group had increased OPG expression compared to the CIA group (Figure 3).

### Western Blot Analyses of *in vivo* Samples

**FIGURE 4:**
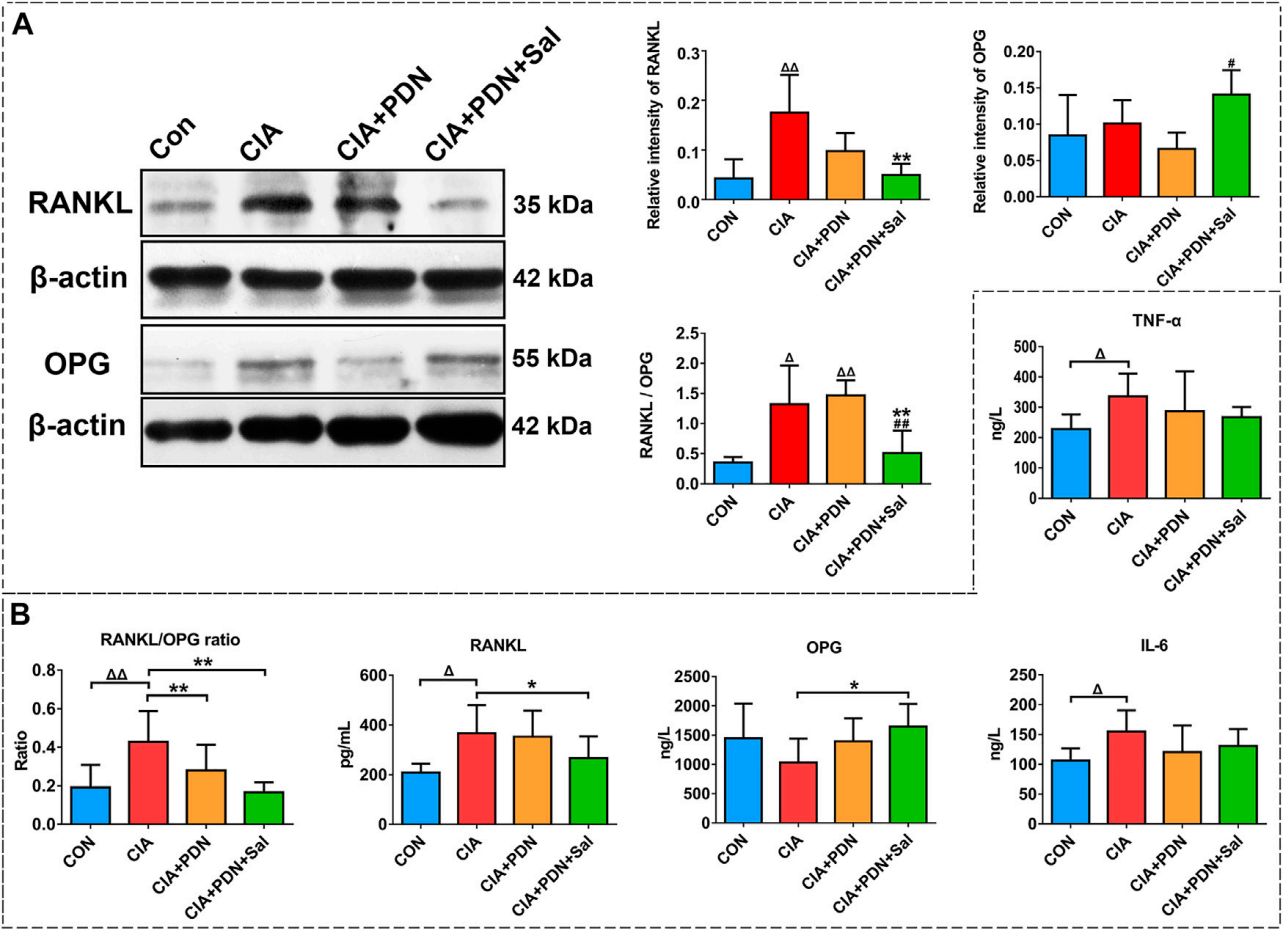
Western blot analysis of RANKL and OPG expression in the hind ankle **(A)** and serum biomarker analysis **(B)** in different groups of rats. ^ΔΔ^ *p* < 0.01 vs. CON, **p < 0.01 vs. CIA, ^#^ *p* < 0.05 vs. CIA+PDN (*n* = 7).

The results of femur western blotting analysis showed that the expression of RANKL and the RANKL/OPG ratio in CIA rats were significantly increased by 308 and 276%, respectively, while the expression of OPG was not significantly changed. The expression of RANKL and the RANKL/OPG ratio were significantly decreased by 72 and 49%, respectively, compared to that in the CIA group. CIA+PND+Sal also significantly increased OPG by 118% and decreased the RANKL/OPG ratio by 65% compared to those in the CIA+PDN group (Figure 4A).

### Serum Biomarker Analyses

Compared with the control group, the levels of IL-6, TNF-α, RANKL and the RANKL/OPG ratio were significantly increased in the CIA group. CIA model induction caused a decrease in OPG expression in the serum. CIA+PDN and CIA+PDN+Sal decreased serum IL-6 and TNF-α levels compared to those in the CIA group. CIA+PDN+Sal significantly decreased sRANKL and sRANKL/OPG and increased OPG levels in serum compared to those in the CIA group (Figure 4B).

### RT-PCR and Western Blot Analyses of *in vitro* Samples

**FIGURE 5:**
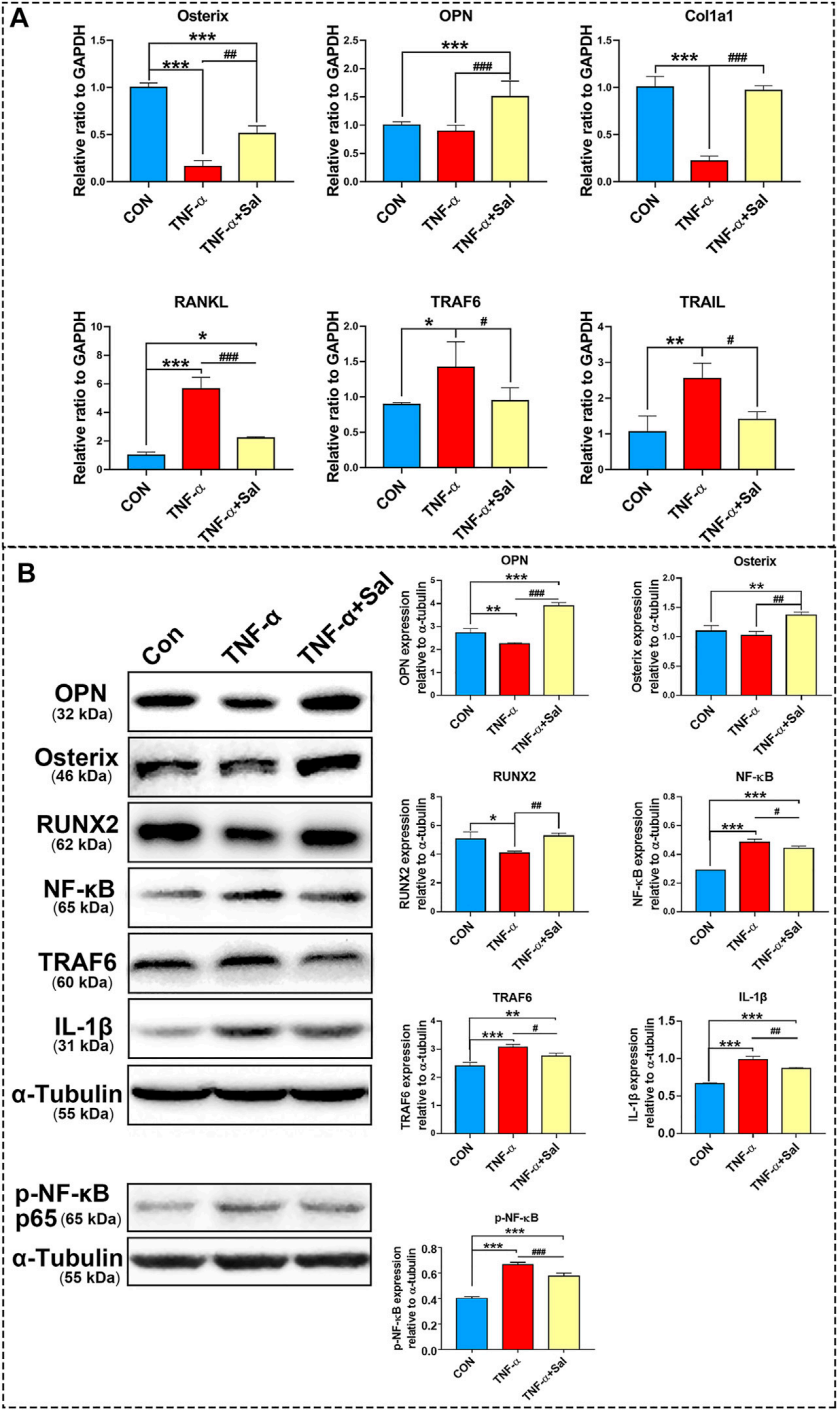
The impacts of salvianolate treatment on TNF-α-induced MC3T3-E1 cells. **(A)** Osterix, OPN, Col1a1, RANKL, TRAF6, and TRAIL gene expression in TNF-α-induced MC3T3-E1 cells after salvianolate treatment based on RT-PCR analyses. **(B)** OPN, RUNX2, NFκB, TRAF6, IL-1β, and p-NF-κB p65 protein expression in TNF-α-induced MC3T3-E1 cells after salvianolate treatment based on western blot analyses. *p < 0.05, **p < 0.01, ***p < 0.001 vs. CON, ^#^ *p* < 0.05, ^##^ *p* < 0.01, ^###^ *p* < 0.001 vs. TNF-α.

RT-qPCR results demonstrated that TNF-α-induced inflammatory medium significantly decreased Osterix, OPN, and Col1a1 expression and increased RANKL, TRAF6, and TRAIL gene expression in MC3T3-E1 osteoblasts compared to that in the control. Salvianolate treatment significantly upregulated Osterix, OPN, and Col1a1 gene expression and downregulated RANKL, TRAF6 and TRAIL gene expression in TNF-α-induced MC3T3-E1 osteoblasts compared to that in the TNF-α-induced group (Figure 5A).

The results of western blotting revealed that TNF-α significantly decreased OPN and RUNX2 protein expression and increased NFκB, TRAF6, and IL-1β protein expression. Salvianolate treatment significantly increased OPN, Osterix, and RUNX2 protein expression and decreased NFκB, TRAF6, and IL-1β protein expression compared to that in the TNF-α-induced group (Figure 5B).

## Discussion

A previous report revealed that 24.8% of female RA patients developed osteoporosis after an average follow-up of 7.4 years after diagnosis (Hwang et al., 2017). There is a large amount of evidence suggesting that accelerated BMD loss occurred in the hands, spine and femoral neck of RA patients compared to that in healthy individuals (Guler-Yuksel et al., 2010; Fassio et al., 2016). Our data showed that CIA induced significant joint destruction and inflammation, and systemic osteoporosis was also found in CIA rats with trabecular and cortical bone degradation, BMD reduction, and bone biomechanical deterioration (Figures 1–3). In our previous study, we found that CIA rats developed systemic osteoporosis along with CIA development (Wu et al., 2016). These results are consistent with previous clinical reports on RA (Kim et al., 2010; Hoes et al., 2015). Previous studies indicated that inflammatory cytokines (such as TNF-α, IL-1β, and IL-6) significantly promoted osteoclastogenesis (Lam et al., 2000; Moon et al., 2013; Wu et al., 2017; Shiratori et al., 2018). In this study, TNF-α and IL-6 in the serum were significantly increased in CIA rats compared to that in normal control rats. Our data indicated that bone resorption (including parameters of Oc. N, %Oc.S.Pm, and RANKL in the serum and femur) was significantly enhanced in CIA rats compared to that in healthy controls (Figures 2C, 3, 4). In addition, our data also demonstrated that the bone formation rate and osteoblast number parameter in the PTM was significantly increased in the CIA group compared to the normal control group (Figure 2C). Interestingly, we noticed that periosteal bone formation did not show a significant difference among the groups; however, endocortical bone formation rates significantly increased in the CIA group compared to the other groups (Figure 3B). These results suggest that RA induced high bone turnover in the trabecular and endocortical regions and that elevated bone formation was insufficient to compensate for the higher bone resorption, ultimately resulting in bone loss.

Glucocorticoid (GC) therapy is a main treatment strategy in RA because of its ability to effectively manage inflammation and reduce disease progression in RA (Bakker et al., 2012; Engvall et al., 2013). In this study, GC therapy effectively controlled progressive inflammation in CIA rats (Figure 4). However, some reports have suggested that long-term, high-dose or even low-dose GC therapy in RA accelerated bone loss and led to a high risk of fractures (Engvall et al., 2008; Wang et al., 2020). A previous study demonstrated that the BMD of the spine and femoral neck continued to decrease after GC therapy in RA patients and that subsequent GC dose adjustments did not help to ameliorate the decline in BMD (Fassio et al., 2016). A high dose of daily GCs *via* systemic administration was associated with elevated fracture risk in RA patients (Balasubramanian et al., 2016). In this study, compared to the CIA group, the CIA+PDN group had significantly suppressed the elevated osteogenesis and osteoclastogenesis and decreased the high bone turnover induced by RA. Prednisone treatment partially alleviated the bone loss in trabecular and trabecular bone microstructure deterioration induced by RA, which may have been due to its effective control of inflammation and the decreased in the high bone turnover of RA. However, prednisone treatment still significantly induced cortical bone loss and increased cortical bone marrow area in CIA rats (Figure 1D). These results suggest that prednisone monotherapy partially ameliorates joint damage and bone loss in trabecular, but still induces cortical bone loss and does not improve bone quality or reduce fracture risk in CIA rats.

In this study, our data demonstrated that CIA+PDN+Sal significantly increased BMD and biomechanical properties, ameliorated bone loss in both trabecular and cortical bone and improved trabecular microstructure compared to those in the CIA and CIA+PDN groups, which suggests that salvianolate treatment significantly improved bone quality. Our previous study demonstrated that salvianolate treatment significantly decreased bone loss in prednisone-treated lupus mice (Liu et al., 2016). In this study, salvianolate significantly inhibited osteoclast number and activity (Oc.N and %Oc.S.Pm), and bone resorption decreased accordingly. RANKL and the RANKL/OPG ratio significantly decreased in the serum, femur and ankle of the CIA+PDN+Sal group, while the serum level of OPG increased significantly.

In addition, we found that salvianolate did not adequately improve bone formation in prednisone-treated CIA rats compared to prednisone treatment alone according to bone histomorphometric analysis. These results were different from those of a previous study in which salvianolic acid B (one of the main bioactive components in salvianolate) promoted the osteogenesis of bone marrow stromal cells and human mesenchymal stem cells *in vitro* (Xu et al., 2014) and increased bone formation by promoting osteoblastic differentiation and bone matrix mineralization in zebrafish larvae (Luo et al., 2016). This may be partly because RA induced significantly increased bone formation with high bone turnover compared to that in the normal control, and salvianolate did not adequately increase bone formation. In addition, prednisone treatment may provide some suppression of bone formation (Rauch et al., 2010; Chotiyarnwong and McCloskey, 2020) to partially block the effects of salvianolate in prednisone-treated CIA rats. Salvianolate showed some synergistic anti-inflammatory effects with prednisone treatment (serum TNF-α). However, the synergistic effect did not reveal a significant difference compared to prednisone treatment alone. These results suggest that combination therapy with prednisone and salvianolate ameliorated bone loss mainly by inhibiting bone resorption through the regulation of the RANKL/RANK/OPG signaling pathway. Previous studies demonstrated that the main bioactive components of salvianolate (salvianic acid A and salvianic acid B) increased the expression of OPG in GC-induced osteoporosis rats (Cui et al., 2009; Cui et al., 2012). In addition, salvianolate ameliorated bone loss in prednisone-treated lupus mice partially through the RANK/RANKL/OPG signaling pathway (Liu et al., 2016). The results of this study were consistent with those of previous reports and indicated that combination therapy with prednisone and salvianolate was superior to prednisone treatment alone for ameliorating systemic bone loss and joint damage in RA rats.

*In vivo* results suggested that RANKL overexpression and OPG suppression in CIA rats and salvianolate treatment could suppress RANKL, increase OPG, and decrease RANKL/OPG ratio. First, we wondered where the OPG and RANKL came from? OPG is a glycoprotein secreted by bone marrow stromal cells (BMSCs) and osteoblasts. OPG acts as an effective inhibitor of osteoclast differentiation, activation and survival and therefore inhibits bone resorption. RANKL is an important cytokine secreted by osteoblasts and their immature precursors, T lympho-cytes, B lymphocytes and megakaryocytes and endothelial cells. RANKL/RANK/OPG is an important osteoclastogenesis signaling pathway that regulates bone resorption (Kong et al., 2000; Thirunavukkarasu et al., 2000; Buckley and Fraser, 2002; Wright et al., 2009; Fumoto et al., 2014; Lee et al., 2018).

To further evaluate the effects of salvianolate under inflammatory conditions, we investigated the effects of salvianolate in MC3T3-E1 osteoblast in this study, as osteoblast secreted both OPG and RANKL to regulate osteoclast activity. The role of osteoblast is important in regulating osteoclast function and activity.

The results demonstrated that salvianolate significantly downregulated *RANKL, TRAF6*, and *TRAIL* gene expression a TNF-α-induced inflammatory osteoblast model. In addition, salvianolate significantly upregulated the gene expression of osteogenic markers (Osterix, OPN, and Col1a1). The results further revealed that salvianolate significantly suppressed the protein expression of IL-1β, NF-κB, and TRAF6 and promoted the protein expression of osteogenic markers (Osterix, OPN, and RUNX2) (Figure 5). TRAF6 is a downstream protein of RANKL. The decrease in TRAF6 further downregulated the NF-κB signaling pathway. The suppression of the RANKL/TRAF6/NF-κB signaling axis and inflammatory cytokine inhibition contributed to osteoclastogenesis suppression. In addition, salvianolate also downregulated TRAIL levels compared to those in the TNF-α-induced group. Decreased TRAIL expression was reported to decrease the apoptosis of osteoblasts (Mori et al., 2009), inhibit RANK signaling and suppress osteoclast activation (Yen et al., 2008; Yen et al., 2012).

**FIGURE 6:**
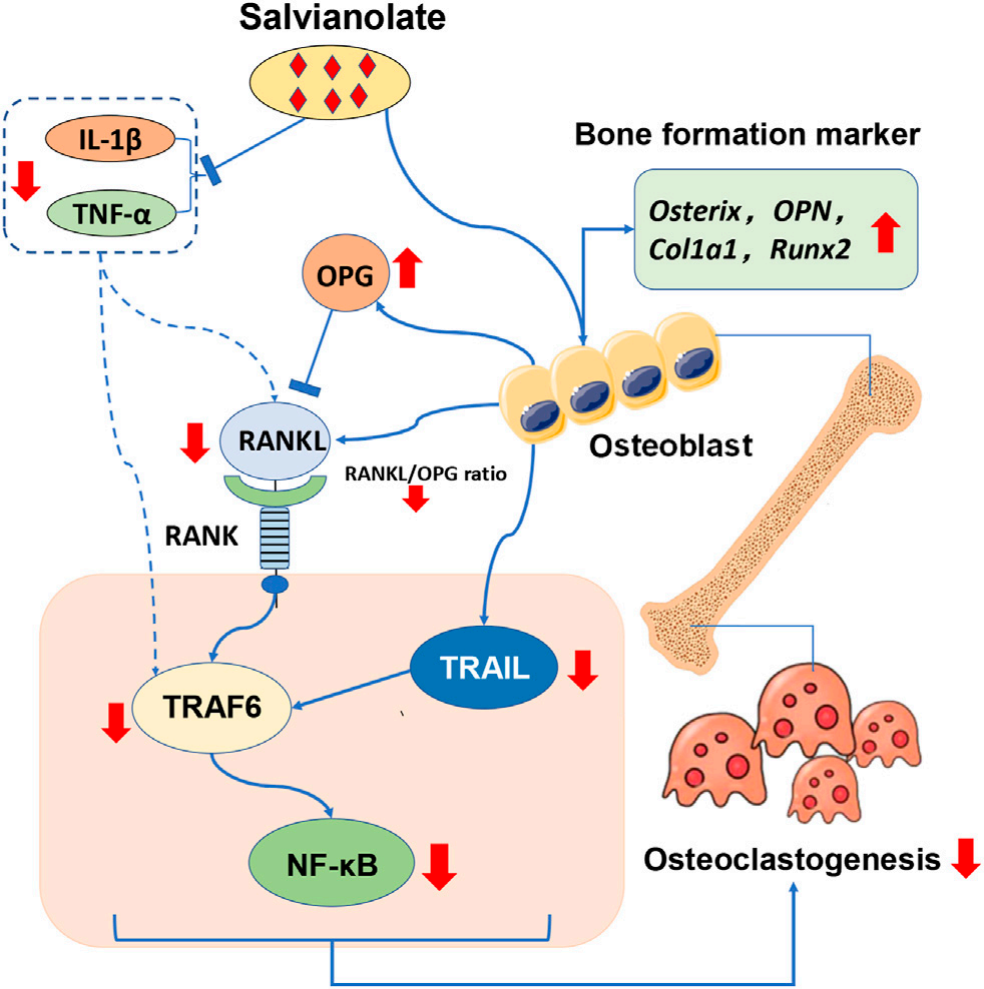
The potential mechanism by which salvianolate ameliorates bone loss in GC-treated RA-OP.

*In vitro* study, we confirmed that osteoblast involves in the regulation of the overexpression RANKL and TRAIL-TRAF6-NFκB signal axis was further activated to stimulate osteoclastogenesis *in vitro*. Salvianolate treatment significant increased osteogenesis (Ostetix, OPN, and Col1a1) and suppress RANKL/RANK/OPG signaling pathway and TRAIL-TRAF6-NFκB signal axis *in vitro* (Figure 6). RANKL and inflammatory cytokines play a key role in the pathogenesis of joint damage and systemic bone mass loss during RA. Previous studies have suggested that the RANKL/RANK/OPG signaling pathway plays a critical role in joint and bone destruction in the pathology of RA. A close relationship among inflammatory cytokines and the NF-κB and RANKL/RANK/OPG signaling pathways has been reported (Zhang et al., 2001; Popova et al., 2019; Ilchovska and Barrow, 2021). It was reported that RANKL-targeted peptides inhibited osteoclastogenesis and attenuated adjuvant-induced arthritis by inhibiting RANKL activation and downregulating inflammatory cytokines (Naidu et al., 2013). In this study, salvianolate inhibited RANKL/RANK/OPG activation, suppress TRAIL-TRAF6-NFκB signal axis, downregulated inflammatory cytokines, and promoted osteogenesis under inflammatory conditions *in vitro*. Due to the high bone turnover of CIA rats and the potent anti-inflammatory effects of GC, salvianolate did not have significant osteogenic and anti-inflammatory effects *in vivo*; however, salvianolate significantly inhibited bone resorption and improved bone quality in the CIA+PDN+Sal group compared to that in the CIA+PDN group *in vivo*.

In this study, the effect of Salvianolate on CIA/GC-treated rat model is more based on bone resorption inhibition other than the stimulation of bone formation. The effects of salvianolate on bone resorption inhibition may due to salvianolate suppress osteoblast-mediated RANKL/OPG signal and TRAIL-TRAF6-NFκB signal axis.

There is a limitation in this study, osteoclast is an important cell in CIA and it is currently unknown whether salvianolate have a direct effect on osteoclast in CIA/GC-treated CIA rats and the underlying mechanism. Further studies are needed to investigate on this point.

In conclusion, combination therapy with prednisone and salvianolate was superior to prednisone monotherapy for ameliorating joint damage and improved bone quality in CIA rats through the regulation of the RANKL/RANK/OPG signaling pathway and TRAIL-TRAF6-NFκB signal axis, thus reducing fracture risk. Salvianolate demonstrated significant osteoclastogenesis suppression, ameliorates osteopenia and improves bone quality, which could be used as a promising antiosteopenia therapeutic strategy to improve bone quality in RA patients.

## Data Availability

The original contributions presented in the study are included in the article/Supplementary Material, further inquiries can be directed to the corresponding author/s.
