# Supplementary material for: Salvianolate Ameliorates Osteopenia and Improves Bone Quality in Prednisone-Treated Rheumatoid Arthritis Rats by Regulating RANKL/RANK/OPG Signaling
Source: Front Pharmacol. 2021 Sep 6;12:710169. doi: 10.3389/fphar.2021.710169 (PMC8450458; doi:10.3389/fphar.2021.710169)
Supplement: Supplementary file 1 [file DataSheet1.docx]

Supplementary materials

According to the manufacturer’s instruction, one unit salvianolate injection (50mg) contains 40mg salvianolic acid B magnesium salt (Magnesium Lithospermate B, CAS.No:122021-74-3, Figure S1).


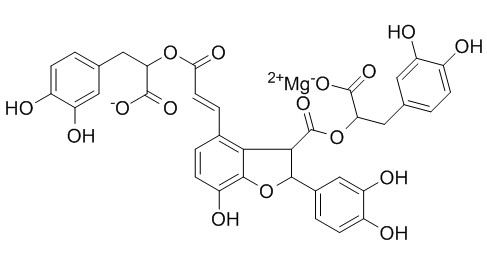


Figure S1 Salvianolic acid B magnesium salt

(Magnesium Lithospermate B, CAS.No:122021-74-3)


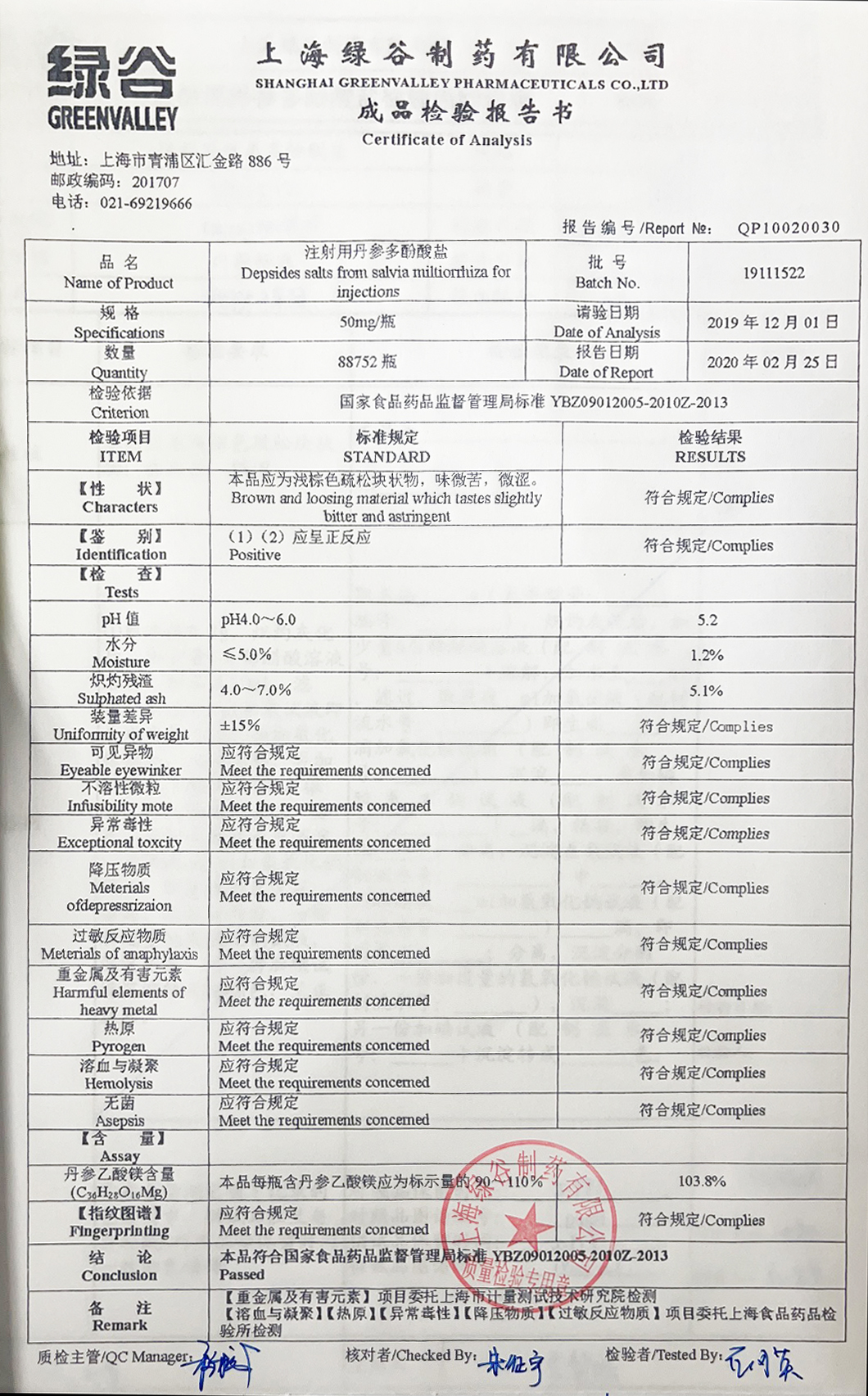


Figure S2 The certificate of analysis of Salvianolate injection


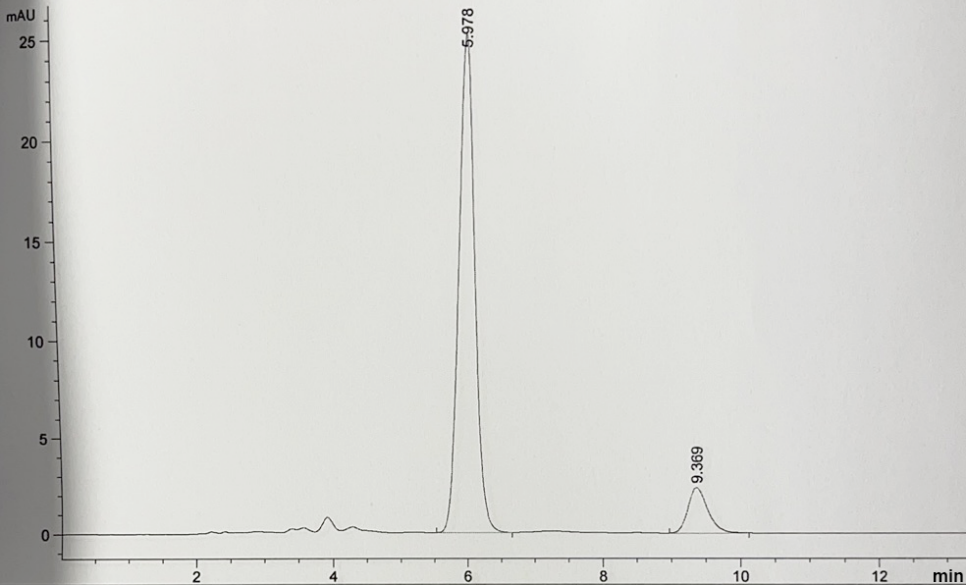


Figure S3 The HPLC analysis of salvianolate.
